# Characterization of the biological activity of a potent small molecule Hec1 inhibitor TAI-1

**DOI:** 10.1186/1756-9966-33-6

**Published:** 2014-01-09

**Authors:** Lynn YL Huang, Ying-Shuan Lee, Jiann-Jyh Huang, Chia-chi Chang, Jia-Ming Chang, Shih-Hsien Chuang, Kuo-Jang Kao, Yung-Jen Tsai, Pei-Yi Tsai, Chia-Wei Liu, Her-Sheng Lin, Johnson YN Lau

**Affiliations:** 1 Taivex Therapeutics Corporation, 17th Floor, No. 3, Yuanqu Street, Nangang District, Taipei City 115, Taiwan; 2 Development Center for Biotechnology, 101, Lane 169, Kangning Street, Xizhi District, New Taipei City 22180, Taiwan

**Keywords:** Hec1, NDC80, Anti-cancer drug, Therapeutics, Mitosis, Apoptosis, P53, Retinoblastoma gene, Markers for response

## Abstract

**Background:**

Hec1 (NDC80) is an integral part of the kinetochore and is overexpressed in a variety of human cancers, making it an attractive molecular target for the design of novel anticancer therapeutics. A highly potent first-in-class compound targeting Hec1, TAI-1, was identified and is characterized in this study to determine its potential as an anticancer agent for clinical utility.

**Methods:**

The *in vitro* potency, cancer cell specificity, synergy activity, and markers for response of TAI-1 were evaluated with cell lines. Mechanism of action was confirmed with western blotting and immunofluorescent staining. The *in vivo* potency of TAI-1 was evaluated in three xenograft models in mice. Preliminary toxicity was evaluated in mice. Specificity to the target was tested with a kinase panel. Cardiac safety was evaluated with hERG assay. Clinical correlation was performed with human gene database.

**Results:**

TAI-1 showed strong potency across a broad spectrum of tumor cells. TAI-1 disrupted Hec1-Nek2 protein interaction, led to Nek2 degradation, induced significant chromosomal misalignment in metaphase, and induced apoptotic cell death. TAI-1 was effective orally in *in vivo* animal models of triple negative breast cancer, colon cancer and liver cancer. Preliminary toxicity shows no effect on the body weights, organ weights, and blood indices at efficacious doses. TAI-1 shows high specificity to cancer cells and to target and had no effect on the cardiac channel hERG. TAI-1 is synergistic with doxorubicin, topotecan and paclitaxel in leukemia, breast and liver cancer cells. Sensitivity to TAI-1 was associated with the status of RB and P53 gene. Knockdown of RB and P53 in cancer cells increased sensitivity to TAI-1. Hec1-overexpressing molecular subtypes of human lung cancer were identified.

**Conclusions:**

The excellent potency, safety and synergistic profiles of this potent first-in-class Hec1-targeted small molecule TAI-1 show its potential for clinically utility in anti-cancer treatment regimens.

## Background

Drugs that interfere with mitosis are part of the most successful cancer chemotherapeutic compounds currently used in clinical practice [1]. Development of chemotherapeutic drugs that target the mitotic cycle has focused on inhibition of the mitotic spindle through interactions with microtubules [1]. Drugs targeting microtubules such as taxanes and vinca alkaloids are effective in a wide variety of cancers, however, the hematopoietic and neurological toxicities as well as development of resistance to this class of drugs severely limit their long term clinical utility [1,2]. Novel anti-mitotic agents have been designed to target the mitotic apparatus through non-microtubule mitotic mediators such as mitotic kinases and kinesins [2].

A novel attractive non-microtubule target is Highly Expressed in Cancer 1 (Hec1), a component of the kinetochore that regulates the spindle checkpoint. Hec1 is of particular interest because of its association with cancer progression [3-5]. Hec1 directly interacts with multiple kinetochore components including Nuf2, Spc25, Zwint-1, and with mitotic kinases Nek2 and Aurora B [6,7] and its expression is tightly regulated in both normal cells and transformed cells during the cell cycle [4,8]. Rapidly dividing cells express a high level of Hec1, in contrast to very low to undetectable levels of Hec1 in terminally differentiated cells [3]. Hec1 has been demonstrated to overexpress in various human cancers including the brain, liver, breast, lung, cervical, colorectal and gastric cancers [3,9]. From a mechanistic standpoint, targeted inhibition of Hec1 by RNAi or by small molecules effectively blocks tumor growth in animal models [3,10]. Therefore, Hec1 emerges as an excellent target for treating cancer clinically.

Small molecules targeting the Hec1/Nek2 pathway was first discovered by Drs. Chen in the laboratory of Dr. W.H. Lee using the inducible reverse yeast two-hybrid screening of a library of ~24,000 compounds [3]. A series of compounds was designed based on this published initial hit molecule as the starting template to optimize the potency for drug development (Huang *et al.*, manuscript in preparation). The original template with micromolar *in vitro* potency was improved to low nanomolar potency, enabling possible clinical utility of the Hec1-targeted compound. This study explores the features and potential of the improved anticancer agent targeting Hec1, TAI-1, for preclinical development and clinical utility. The *in vitro* and *in vivo* biological activity, mechanism of action, toxicity and safety, and translational implications are investigated.

## Methods

### Cell lines

MDA-MB-231, MDA-MB-468, K562, HeLa, MCF7, HCC1954, A549, COLO205, U2OS, Huh-7, U937, HepG2, KG-1, PC3, BT474, MV4-11, RS4;11, MOLM-13, WI-38, HUVEC, RPTEC, and HAoSMC were from Development Center for Biotechnology, New Taipei City, Taiwan; MDA-MB-453, T47D, ZR-75-1, ZR-75-30, MDA-MB-361, Hs578T, NCI-H520, Hep3B, PLC/PRF/5 were from Bioresource Collection and Research Center, Hsinchu, Taiwan. Cell lines were maintained in complete 10% fetal bovine serum (Biowest, Miami, FL, USA or Hyclone, Thermo Scientific, Rockford, IL, USA) and physiologic glucose (1 g/L) in DME (Sigma, St. Louis, MO, USA). Studies conducted using cell lines RPMI8226, MOLT-4, and N87; drug-resistant cell lines MES-SA/Dx5, NCI/ADR-RES, and K562R were from and tested by Xenobiotic Laboratories, Plainsboro, NJ, USA.

### *In vitro* potency assay

Cells were seeded in 96 well plates, incubated for 24 hours, compounds added and incubated for 96 hours. All testing points were tested in triplicate wells. Cell viability was determined by MTS assay using CellTiter 96® Aqueous Non-radioactive Cell Proliferation Assay system (Promega, Madison, WI, USA) according to manufacturer’s instructions with MTS (Promega) and PMS (Sigma, St. Louis, MO). Data retrieved from spectrophotometer (BIO-TEK 340, BIOTEK, VT, USA) were processed in Excel and GraphPad Prism 5 (GraphPad Software, CA, USA) to calculate the concentration exhibiting 50% growth inhibition (GI_50_). All data represented the results of triplicate experiments.

### Immunoblot and co-immunoprecipitation analysis

Western blotting and co-immunoprecipitation were done as described previously [3]. Primary antibodies used: mouse anti-Nek2 and mouse anti-Mcl-1 (BD Pharmingen, San Diego, CA); rabbit anti-Hec1 (GeneTex, Inc., Irvine, CA); mouse anti-actin (Sigma); mouse anti-P84 and mouse anti-RB (Abcam, Cambridge, MA); rabbit anti-Cleaved Caspase3, rabbit-anti-Cleaved PARP, rabbit anti-XIAP, and mouse anti-P53 (Cell Signaling Technology, Boston, MA); mouse anti-Bcl-2 (Santa Cruz); mouse anti*-α-*Tubulin (FITC Conjugate; Sigma).

For co-immunoprecipitation, cells were lysed in buffer (50 mM Tris (pH 7.5), 250 mM NaCl, 5 mM EDTA (pH 8.0), 0.1% Triton X-100, 1 mM PMSF, 50 mM NaF, and protease inhibitor cocktail (Sigma P8340)) for 1 hour then incubated with anti-Nek2 antibody (rabbit, Rockland) or IgG as control (rabbit, Sigma-Aldrich, St. Louis, MO) for 4 hours at 4°C, collected by protein G agarose beads (Amersham) and processed for immunoblotting.

### Immunofluorescent staining and microscopy

For quantification of mitotic abnormalities, cells were grown on Lab-Tek® II Chamber Slides, washed with PBS buffer (pH 7.4) before fixation with 4% paraformaldehyde. Following permeabilization with 0.3% Triton X-100, cells were blocked with 5% BSA/PBST and incubated with anti*-α-*Tubulin antibodies. Then DAPI (4’,6’-diamidino-2-phenylindole) staining was applied and cells were mounted with ProLong® gold antifade (Life Technologies). Images were examined with NIKON 80i microscope at 400× or 1000x magnification and captured with Spot Digital Camera and Spot Advanced Software Package (Diagnostic Instruments, Sterling Heights, MI). The percentage of cells with mitotic abnormalities was calculated by the number of the cells showing the abnormal mitotic figures (including chromosomal misalignment and formation of multipolar spindles) divided by the total number of mitotic cells counted. A minimum of 500 cells from randomly selected fields were scored per condition per experiment.

### Mouse xenograft model

The procedure was adapted from published protocol [3] and were in accordance to the Institutional Animal Care and Use Committee of DCB. C.B-17 SCID mice (6-7 weeks, 21-24 g) (Biolasco, Taipei, Taiwan) were used. Females were used for Colo-205 and Huh-7 while and males were for MDA-MB-231. Cells were injected subcutaneously into the flank in 50% matrigel solution (BD Biosciences, San Jose, CA). 1×10^7^, 3×10^6^, and 6×10^6^ implanted cells/mouse was used for Huh-7, Colo-205, and MDA-MB-231, respectively. Treatment initiated when tumor volume reached 150 mm^3^. For Colo-205 and Huh-7, mice were treated with vehicle control (10% DMSO 25% PEG200) per oral PO/BID/28 cycles in total. For Huh-7, a dose increase was incurred on day 4 to increase efficacy. For Colo205, a dose decrease was incurred on day 13 to decrease body weight loss. For MDA-MB-231, mice were treated with vehicle control (5% DMSO, 10% Cremophor, 85% water for Injections (WFI)) per oral PO/BID/28 cycles in total, or TAI-1 formulated in vehicle (20 mg/kg intravenously IV/QDx28 cycles or 150 mg/kg per oral PO/BID/28 cycles in total). Tumor size were measured with digital calipers and volume calculated using the formula (L x W x W)/2, of which L and W represented the length and the width in diameter (mm) of the tumor, respectively. Body weights and tumor growth were measured twice a week. Mean tumor growth inhibition of each treated group was compared with vehicle control and a tumor growth inhibition value calculated using the formula: [1-(T/C) ×100%] (T: treatment group, C: control group tumor volume).

### Pilot toxicology study in mice

A sub-acute toxicology study was performed for TAI-1. Female C.B-17 SCID mice (7 weeks old) were used in this study. Mice were divided into four treatment groups: vehicle control (10% DMSO, 25% PEG200, 65% double distilled H_2_O), test article (in vehicle) at 7.5, 22.5, and 75.0 mg/kg, and all mice were treated twice a day by oral administration for 7 days (n = 8 for each group). Body and organ weights were measured. Blood were collected by cardiac puncture and serum analyzed for complete blood count and biochemical indices.

### *In vitro* kinase assay

Inhibition of kinase activity by test compound was estimated by [^33^P] labeled radiometric assay. 20 kinase assays (Millipore) were adapted. The kinase reaction was performed according to individual manual with minor modification. In brief, each test compound was evaluated at two concentrations (10 mM and 1 mM) in duplication. The kinase reaction were initiated by enzyme addition, stopped at indicated time by the addition of 3% phosphoric acid, harvested onto a filter plate by using a unifilter harvester (PerkinElmer), and counted by using TopCount (PerkinElmer). The results were the average of duplicate measurements and expressed as percentage inhibition (compound treatment versus DMSO control).

### Cardiac toxicology study - hERG binding assay

[^3^H]Astemizole competitive binding assays are performed to determine the ability of compounds to displace the known radioligand [^3^H]-astemizole from the hERG potassium channels, following standard protocol with minor modifications. In brief, assays were performed in 200 μl of binding buffer (50 mM HEPES, pH 7.4, 60 mM KCl, and 0.1% BSA) containing 1.5 nM of [^3^H]astemizole, 3 μg/well of hERG membrane protein (PerkinElmer), and TAI-1 (in 1% DMSO final concentration) at 27°C for 60 min. Nonspecific binding (NSB) was determined in the presence of 10 μM astemizole. IC_50_ assay for TAI-1 contained 8 concentration points with 10-fold serial dilution in triplicate. Binding was terminated by rapid filtration onto polyethyleneimine-presoaked, buffer-washed UniFilter-96, and GF/C (Perkin Elmer) using a vacuum manifold (Porvair Sciences). Captured radiolabel signal was detected using TopCount NXT (Perkin Elmer). The data were analyzed with nonlinear curve fitting software (PRISM, Graphpad) and IC_50_ value (defined as the concentration at which 50% of [^3^H]-astemizole binding is inhibited) was calculated. All results are derived from two independent experiments.

### Drug-drug synergy experiments

Interaction (synergy, additive, antagonistic activities) between Hec1 inhibitor TAI-1 and anticancer drugs (sorafenib, doxorubicin, paclitaxel, and topotecan) were evaluated using standard assays. Twenty-four hours after seeding, cells were treated with TAI-1, the other testing drug, or in combination. For combination testing, TAI-1 or the other testing drugs were added to plate in triplicate wells in ratios of GI_50_ (GI_50A_: GI_50B_), and cells are incubated in drug-treated medium for 96 h and cell viability determined by MTS. Synergy was determined by calculating combination index (CI) value with the formula

$$\mathrm{CI}=\left(C_{A,X}/{\mathrm{IC}}_{X,A}\right)+\left(C_{B,X}/{\mathrm{IC}}_{X,B}\right)$$

where C_A,X_ and C_B,X_ are concentrations of drug A and drug B used in combination to achieve x% drug effect. IC_x,A_ and IC_x,B_ are concentrations for single agents to achieve the same effect. All data represent results of triplicate experiments (and data on mean of three separate determinations had variations of less than ±20%).

### Gene silencing by siRNA transfection

Cells were seeded onto 96-well plates and transfected with siPort NeoFx transfection method (Ambion, Inc., TX, USA) according to manufacturer’s instructions. Cells were cultured for 24 h and treated with compound. SiRNA from two different sources were used to confirm results. At least two independent experiments are used to determine representative results. Control siRNA (#4390843, Ambion, Inc., Austin, TX, USA or #6568 s, Cell Signaling Technology or sc-37007, Santa Cruz Biotechnology), RB siRNA (Silencer Select ID:s523, Ambion or sc-29468, Santa Cruz Biotechnology), and P53 siRNA (#6231 s, Cell Signaling Technology, or sc-29435, Santa Cruz Biotechnology) were employed. The sequences of these control siRNAs are detailed in the manufacturer websites.

### Quantitative real-time RT-PCR

Total RNA was isolated with Quick-RNA miniPrep (Zymo Research, Irvine, CA, USA). Reverse transcription and quantitative real-time PCR was performed on ABI Prism 7500 (PE Applied Biosystems, TX, USA) using the One-Step SYBR ExTaq qRT-PCR kit (Takara, Shiga, Japan) according to manufacturer’s instructions. The following primers were used:

for GAPDH

5′-GGTTTACATGTTCCAATATGATTCCA-3′ (forward), and 5′-ATGGGATTTCCATTGATGACAAG -3′ (reverse);

for RB

5′-GCAGTATGCTTCCACCAGGC-3′ (forward), and 5′-AAGGGCTTCGAGGAATGTGAG-3′ (reverse); and

for P53

5′-GCCCCCAGGGAGCACTA-3′ (forward), and

5′-GGGAGAGGAGCTGGTGTTG-3′ (reverse).

### Gene expression in clinical samples–data from databases

NDC80 (Hec1) gene expression data in non-small cell lung cancer (NSCLC) were retrieved from publicly available database (Gene Expression Omnibus-GSE8894, GSE3141 and GSE37745). Gene expression intensities were normalized with quantile normalization. NDC80 expression between adenocarcinoma and squamous carcinoma was compared for all three different datasets. Eight genes known to associate with NDC80 were identified (18, 27). One way hierarchical clustering analysis for adenocarcinoma and squamous carcinoma of NSCLC was conducted by using R package software (http://www.r-project.org/).

## Results

### Hec1 inhibitor TAI-1 is highly potent with a wide anti-cancer spectrum

**Figure 1:**
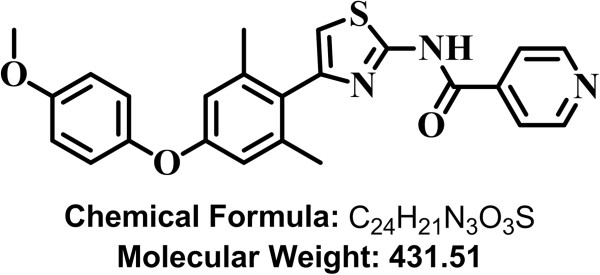
Structure of Hec1 Inhibitor TAI-1.

The initial small molecule hits identified by Drs. Chen in Dr. WH Lee’s laboratory, INH1 and INH2, had micromolar potency on cancer cell lines [3,11,12]. Through medicinal chemical efforts to modify the hit structure, we have significantly improved the potency of the Hec1-targeted compound to low nanomolar level. The new compound, TAI-1, has a GI_50_ of 13.48 nM (K562 cells), which is close to 1000 times improvement in potency compared to INH1 (GI_50_ = 11.7 μM) (14). To characterize the potency of the new compound, TAI-1 (Figure 1), a series of cancer cell lines were tested. The screen includes 31 cancer cell lines, is comprise of 12 cell lines from the NCI-60 panel, and includes breast cancer, leukemia, liver, lung, colon cancer, cervical cancer, prostate cancer and bone cancer with various cellular characteristics. Growth inhibition was quantitated with established MTS assay. As summarized in Table 1, TAI-1 inhibits cellular growth at nM levels for the majority of cancer cell lines screened.

**Table 1:** Characteristics of cell lines screened in Hec1 inhibitor drug assays

	**Cell lines**	**Cell type**	**RB**	**P53**	**GI**_**50 **_**(nM)**
Strong sensitivity (GI_50_ < 50 nM)	K562	Chronic myeloid leukemia	WT	Mut	13
	HeLa	Cervical cancer	Mut	Inactivated	16
	T47D	Breast, metastatic-pleural, invasive ductal carcinoma	WT	Mut	17
	U-937	Acute myeloid leukemia	WT	Null	22
	MDA-MB-453	Breast, metastatic-effusion, adenocarcinoma	WT	Deletion	24
	RPMI8226	Acute myeloid leukemia	Mut	Mut	27
	KG-1	Myelogenous leukemia	Rearranged	Reduced/no	29
	MDA-MB-468	Breast, metastatic-pleural, invasive ductal carcinoma	Mut/no	Mut	34
	HCT116	Colorectal carcinoma	Low	WT	39
	COLO205	Colorectal carcinoma	WT	No	40
	MDA-MB-231	Breast, metastatic-pleural, invasive ductal carcinoma	WT	Mut	43
Moderate sensitivity (50 nM < GI_50_ < 100 nM)	PC3	Prostate cancer	WT	Null	60
	MCF7	Breast, metastatic-pleural, invasive ductal carcinoma	WT	Heterogenous. WT	64
	NCI-H520	Non-small cell lung cancer	WT	Reduced mRNA	68
	ZR-75-30	Breast, metastatic-ascites, invasive ductal carcinoma	WT	WT	77
	ZR-75-1	Breast, metastatic-ascites, invasive ductal carcinoma	WT	WT	80
	Huh7	Hepatocellular carcinoma	WT	Mut	84
	BT474	Breast, primary, invasive ductal carcinoma	WT	Mut	86
	PLC/PRF/5	Hepatocellular carcinoma	WT	Inactivated	92
	Hep3B	Hepatocellular carcinoma	No	Deletion	96
Low sensitivity (100 nM < GI_50_ < 1 μM)	U2OS	Osteosarcoma	Less active	WT	139
	Hs578T	Breast, metastatic, invasive ductal carcinoma	WT	Mut	143
	MV4-11	Acute myeloid leukemia	WT	Mut	231
	RS4;11	Acute myeloid leukemia	WT	Mut	254
	HepG2	Hepatocellular carcinoma	WT	WT	273
	MOLM-13	Acute myeloid leukemia	WT	Mut	315
Resistant (GI_50_ > 1 μM)	A549	Non-small cell lung cancer	WT	WT	>10 μM
	HCC1954	Breast, invasive ductal carcinoma	Mut	WT	>10 μM
	MDA-MB-361	Breast, metastatic-brain, adenocarcinoma	WT	No	>10 μM
	MOLT-4	Acute lymphoblastic leukemia	WT	WT	>30 μM
	N87	Gastric cancer	WT	WT	>30 μM

*WT, wild type; Mut, mutated.

To determine the activity of TAI-1 in multidrug resistant (MDR) cell lines, established MDR cell lines were tested. MES-SA/Dx5 and NCI-ADR-RES are resistant to doxorubicin and paclitaxel, while K562R cells are resistant to imatinib. TAI-1 was active in these cell lines showing nM GI_50_ (Table 2).

**Table 2:** **GI**_**50**_**s of TAI-1 and commerically available drugs in cell lines**

	**Cell line**	**TAI-1 GI**_**50 **_**(nM)**
Drug resistant cancer cell lines	MEX-SA/Dx5	35
	NCI/ADR-RES	29
	K562R	30
Normal cell lines	WI-38	>10 μM
	RPTEC	>10 μM
	HuVEC	> 9 μM
	HAoSMC	> 9 μM

*N.D, not determined.

### TAI-1 targets the Hec1-Nek2 pathway and induces apoptotic cell death

**Figure 2:**
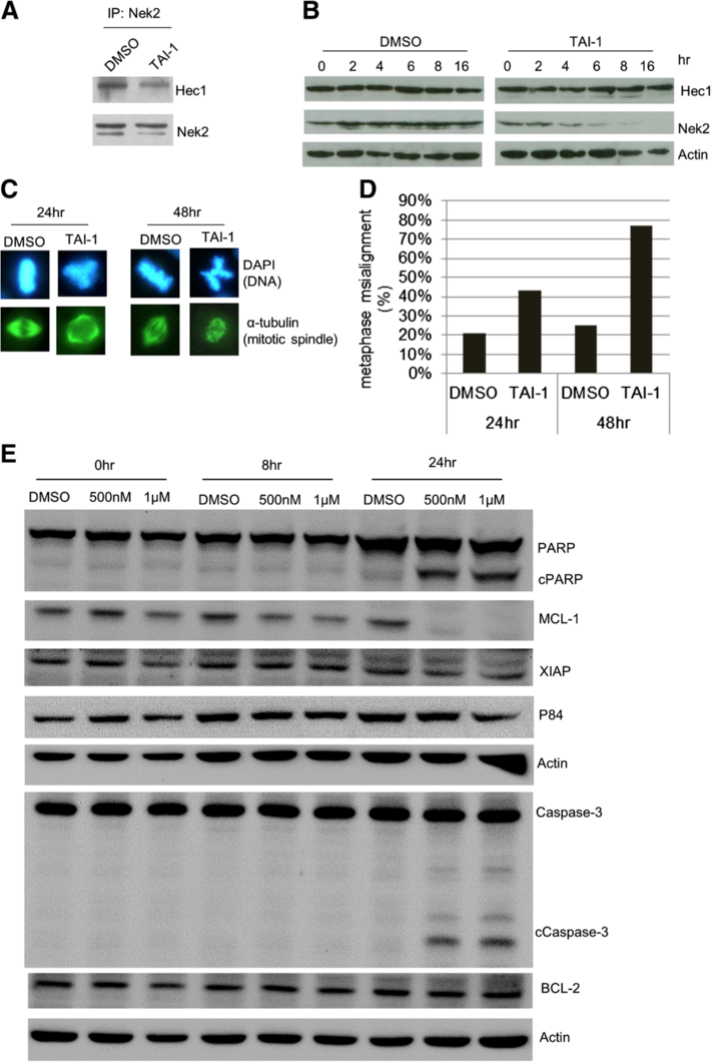
**TAI-1 Disrupts Hec1-Nek2 interactions, induces chromosomal misalignment and induces apoptosis of cancer cells. (A)** K562 cells were treated with 500 nM TAI-1, lysates immunoprecipitated with anti-Nek2 antibody were probed for Hec1 by western blotting to determine interaction. **(B)** K562 cells were treated with TAI-1 at 1 μM for the indicated time points and collected for immunoblotting of Hec1 and Nek2. **(C)** MDA-MB-468 cells treated with 1 μM TAI-1 were immunofluorescent stained for DNA and mitotic spindle. **(D)** Metaphase cells were counted for percentage of cells with misaligned chromosomes. **(E)** Lysates of HeLa treated with TAI-1 for 8 or 24 hours were western blotted for apoptotic markers caspase3 and PARP and anti-apoptotic markers MCL-1, XIAP, and BCL-2. Actin was used as loading control.

To confirm the mechanism of action of TAI-1, we used established methods to evaluate the interaction of Hec1 and Nek2 and the consequences of disruption of interaction of the proteins [3]. Co-immunoprecipitation study shows that TAI-1 disrupted the binding of Nek2 to Hec1 in TAI-1-treated cells (Figure 2A). Disruption of Nek2 binding to Hec1 was shown to lead to degradation of Nek2 [3], and this was also confirmed for TAI-1 (Figure 2B). In addition, previous study also show that disruption of Hec1-Nek2 interaction leads to misaligned chromosomes. Treatment of cells with TAI-1 induced a time-dependent increase in the proportion of cells with chromosomal misalignment in cells (Figure 2C and D). These results are consistent with the phenotypic consequences of the original hit compound INH1 and show that TAI-1 targets Hec1-Nek2 interactions.

The cell death pathway was evaluated with apoptotic markers. Results show that TAI-1 induces cancer cell death through the induction of cleavage of apoptotic proteins Caspase 3 and PARP and degradation of anti-apoptotic proteins MCL-1 and suggests that TAI-1 leads to activation of the apoptotic pathways (Figure 2E).

### TAI-1 effectively inhibits tumor growth in multiple cancer xenograft models

**Figure 3:**
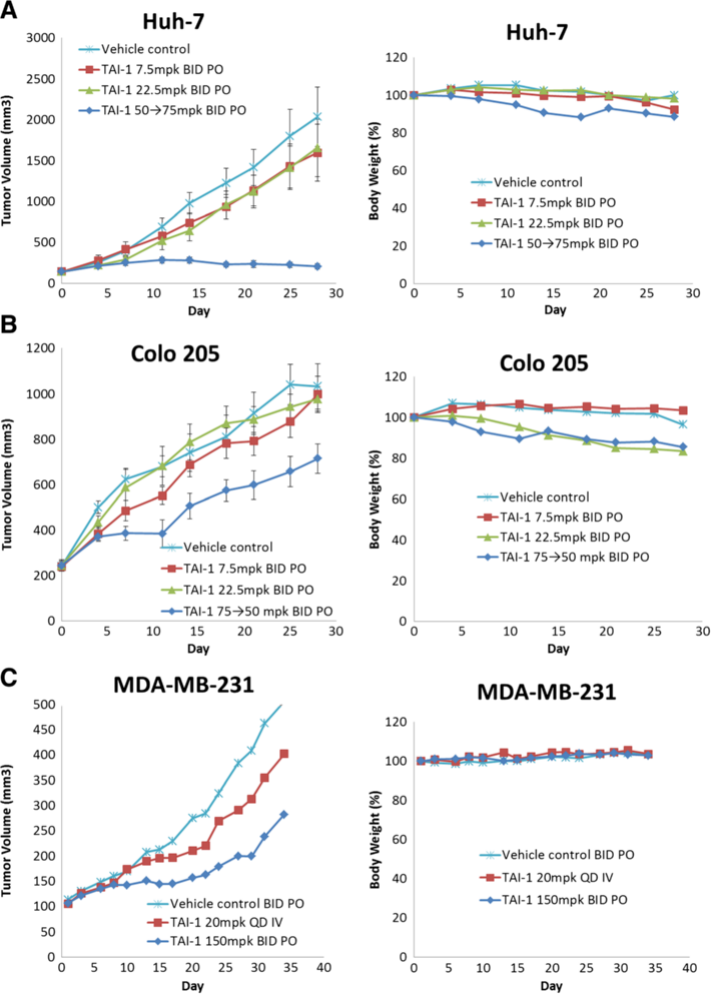
**TAI-1 inhibits growth of multiple tumor types in xenografted mouse models.** Nude mice engrafted with cancer cell lines were treated for 28 days either orally or intravenously as indicated and tumor size measured daily. Huh-7 **(A)**, Colo205 **(B)**, and MDA-MB-231 **(C)** cells were used. Left panel:% tumor inhibition. Right panel:% body weight.

To evaluate the *in vivo* efficacy of TAI-1, xenografted mice models of human tumor cancer cell lines were used. Well-established Huh-7 (hepatocellular carcinoma), Colo205 (colorectal adenocarcinoma from metastasis and ascites), and MDA-MB-231 (triple negative breast cancer cell line) derived models were used. Implanted tumors are allowed to grow to 100-150 mm^3^, then mice were orally administered TAI-1, since the compound was to be developed as an oral drug. TAI-1 led to significant tumor growth retardation in Huh-7 and modest tumor inhibition was noted tor the Colo205 and MDA-MB-231 models (Figure 3 left panels). Intravenous route was also evaluated in MDA-MB-231, but showed a modest effect. Administration of oral and intravenous doses did not lead to any loss in body weight (Figure 3 right panels) or any observed clinical signs.

### Toxicity studies of TAI-1 in rodents

**Figure 4:**
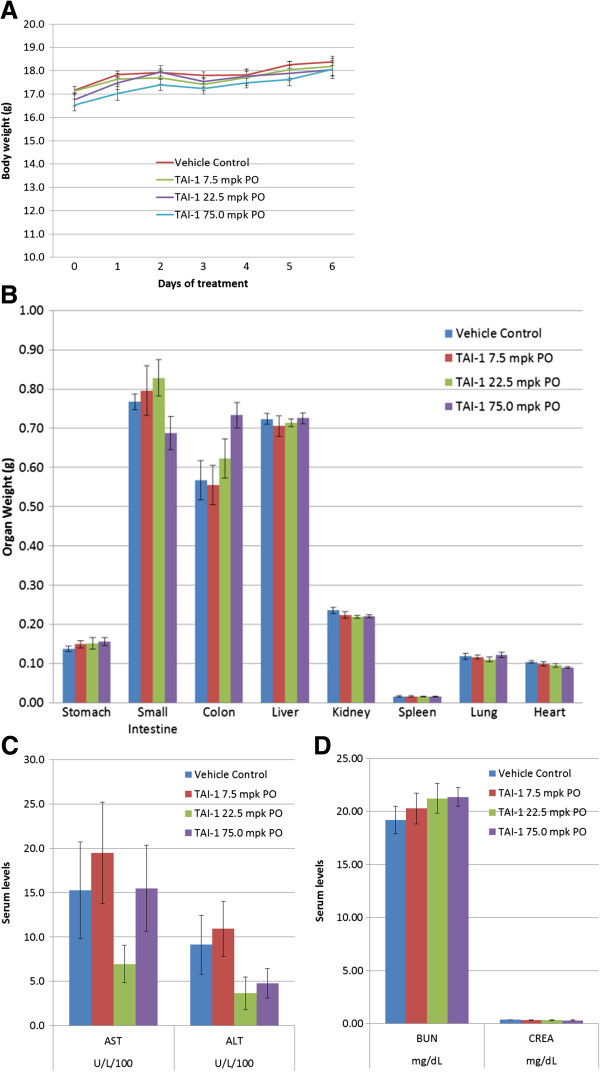
**7-day toxicology study of TAI-1 in mice shows no significant change in body weight, organ weight, and plasma indices.** C.B-17 SCID mice (n = 8) were orally administered TAI-1 for 7 days and body weights **(A)** and organ weights **(B)** were measured. Liver **(C)** and kidney **(D)** plasma indices were determined.

To determine potential toxicity of TAI-1 in orally efficacious treatment regimen, a pilot toxicity study was performed in mice at oral doses corresponding to that used in xenograft studies. The same species and gender of mice were used and dosed at the corresponding doses for 7 days. Daily observation of clinical signs (pain, locomotion/body stature, skin/coat abnormalities) and defecation changes were performed and no changes were noted. Body weight, complete blood count, and serum biochemistry were monitored before and after dosing (Day 0 and Day 7). Postmortem observation of the gastrointestinal tract, liver, kidney, spleen, lung and heart were performed and organ weights were measured. No body weight or organ weight loss was noted (Figure 4A and B). No adverse effects on liver and kidney indices were noted (Figure 4C-D). In addition, no changes in red and white blood cells plasma indices were noted at the efficacy doses tested (Additional file 1: Table S1 and Table S2). TAI-1 shows no adverse effect under efficacious oral dose levels.

### Safety studies of TAI-1

The clinical application of anticancer drugs is often limited by their non-specific target activity leading to organ toxicity and other side effects. To evaluate the preliminary safety profile of TAI-1, we investigated the inhibitory potential of TAI-1 against normal cell lines, against a panel of kinases, and also on its binding to hERG, a known target for cardiac toxicity.

To determine the cancer cell specificity of TAI-1, normal cell lines were tested. In normal fibroblast (WI-38), renal tubule cells (RPTEC), umbilical vein cells (HuVEC) and aortic smooth muscle (HAoSMC) cell lines, TAI-1 had a GI_50_ of more than 1000 times that of cancer cell GI_50_ (Table 2), showing a high therapeutic index.

**Figure 5:**
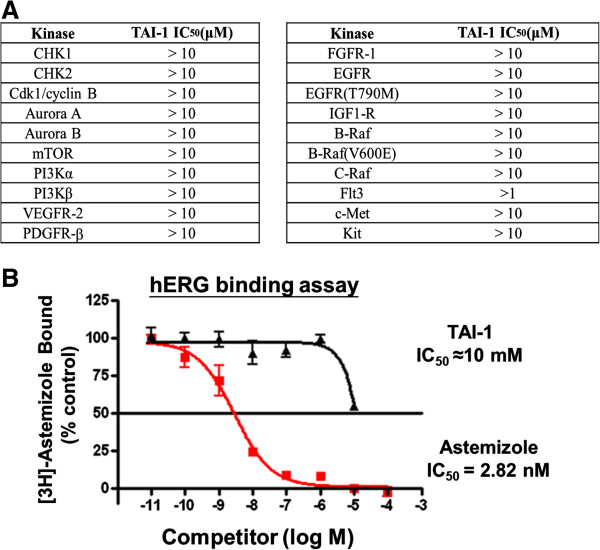
**TAI-1 does not inhibit a number of kinases and hERG at below 10 μM. (A)** Inhibition of kinases were performed with 10 μM TAI-1 with standard assays. **(B)** hERG inhibition was determined with 10 μM TAI-1. Results show good cardiac safety of TAI-1.

When screened against a panel of known kinases, TAI-1 has no inhibitory effects against these targets (Figure 5A), confirming the specificity of TAI-1 to Hec1 and against these kinases targets.

We have tested TAI-1 with the hERG assay, which assesses the most common mechanism involved in drug-induced prolongation of QT interval, which increases the risk of ventricular tachyarrhythmia through the inhibition of potassium ion flow and may lead to sudden cardiac death [13,14]. The hERG channel assay revealed a competition IC_50_ 1000 times that of cancer cell GI_50_ (Figure 5B), suggesting that this compound has little potential of cardiac toxicity through the hERG channel at the therapeutic doses. In summary, TAI-1 exhibits high specificity to cancer cells and to target and shows no cardiac toxicity by hERG.

### TAI-1 is synergistic with some commonly used cytotoxic drugs

Synergy with currently available anti-cancer drugs demonstrates possibility of a compound to be utilized in combinatorial treatment approach. To determine possible synergistic combinations, the effects of TAI-1 in combination with various cytotoxic drugs were evaluated. TAI-1-sensitive cancer cells were treated with an appropriate ratio of doses of cytotoxic agents to TAI-1 determined by corresponding drug GI_50_, as shown in Table 3 (Drug 1: TAI-1 GI_50_ ratio) and MTS assay used to determine cellular proliferation. Combination index (CI) was calculated from the GI_50_s obtained to represent additive (CI = 1), synergistic (CI < 1) or antagonistic (CI > 1) effects. TAI-1 was synergistic with doxorubicin, topotecan, and paclitaxel, but not synergistic with sorafenib and the novel src inhibitor KX-01 [15] (Table 3).

**Table 3:** Synergistic effects of TAI-1 with cytotoxic agents

**Drug**	**Cell lines**	**Drug 1 GI**_**50 **_**(nM)**	**TAI-1 GI**_**50 **_**(nM)**	**Drug 1: TAI-1 GI**_**50 **_**ratio**	**Combination index**	**Synergy**
Doxorubicin	K562	36	44	0.83	0.66	Yes
	MDA-MB-468	27	34	0.80	0.87	Yes
	Huh7	183	84	2.17	0.73	Yes
Topotecan	MDA-MB-231	347	43	8.01	0.78	Yes
	MDA-MB-468	11	34	0.32	0.74	Yes
Paclitaxel	Huh7	94	84	1.11	0.28	Yes
	MDA-MB-231	5	42	0.12	0.68	Yes
	K562	10	41	0.24	0.73	Yes
Sorafenib	Huh7 (liver)	4501	84	53.38	1.66	Antagonistic
	Hep3B (liver)	3676	104	35.50	1.50	Antagonistic
KXO1	Huh7 (liver)	27	84	0.32	1.31	Additive

*Combination index: 1 = additive, < 1 = synergy, > 1 = antagonistic.

### Role of RB and P53 in TAI-1 cellular sensitivity

TAI-1 is active on a wide spectrum of cancer cell lines; however, 5 cell lines were resistant to TAI-1 (Table 1). To explore possible resistance mechanisms of TAI-1, we evaluated the role of retinoblastoma protein RB (a Hec1 interacting protein [4,16] through which Hec1 was discovered), and P53, another oncogene in the same category as RB, which might provide a cellular escape mechanism. The RB and P53 tumor suppressors are both critical players in DNA damage checkpoint [17]. A cross-tabulation comparison of the RB [17-22] and P53 [20,22-28] gene status versus sensitivity to TAI-1 (in this case, response is identified as GI_50_ of < 1 μM, n = 19) revealed an interesting pattern of response to Hec1 inhibitor TAI-1 (Table 1).

**Figure 6:**
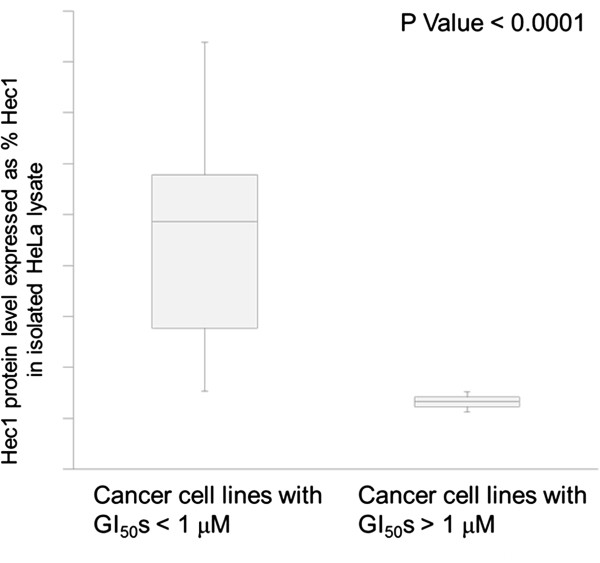
**TAI-1 GI**_**50**_**s correlates with Hec1 protein expression in cancer cell lines.** Asynchronously maintained cell lines are lysed and their total protein immunoblotted for expression levels of Hec1. Hec1 protein expression levels are quantitated and expressed in% relative to HeLa expression levels.

To quantitate Hec1 protein expression levels, we analyzed the expression levels of the Hec1 protein by western blotting and quantitated protein levels using HeLa as standard, and high expression determined as > 50% HeLa expression levels. As shown in Figure 6, cell lines showing a good cellular proliferative response to TAI-1 (as defined by GI_50_ < 1 μM) had a much higher level of expression of Hec1 compared with resistant cell lines (GI_50_ > 1 μM) (p < 0.0001). Table 4 shows the relationship between the expression of Hec1 and the status of the markers. High level expression of Hec1 was associated with a better response to the Hec1 inhibitor TAI-1 (16/16 of High Hec1 expression were sensitive compared to 1/3 of the low Hec1 expression cell lines, p < 0.01).

**Table 4:** Predictive values of biomarkers for Hec1 therapy

**Hec1 expression**				**Hec1 +/- P53 expression**			
	Total	Mut	WT		Total	Mut	WT
Sensitive	17	16	1	Sensitive	25	25	0
Resistant	2	0	2	Resistant	5	1	4
	P value < 0.01				P value < 0.0001		
**P53 expression**				**Hec1 +/- RB expression**			
	Total	Mut	WT		Total	Mut	WT
Sensitive	25	22	3	Sensitive	25	18	7
Resistant	5	1	4	Resistant	5	0	5
	P value < 0.005				P value < 0.005		
**RB expression**				**Hec1 +/- RB +/- P53 expression**			
	Total	Mut	WT		Total	Mut	WT
Sensitive	25	7	18	Sensitive	25	25	0
Resistant	5	0	5	Resistant	5	1	4
	P value = 0.3				P value < 0.0001		
**RB +/- P53 expression**							
	Total	Mut	WT				
Sensitive	25	23	2				
Resistant	5	1	4				
	P value < 0.005						

NOTE: Drug-sensitive (TAI-1 GI_50_ < 300 nM); Drug-resistant (TAI-1 GI_50_ > 300 nM); Mut (high Hec1 protein expression level (> 50% HeLa expression), mutated/aberrant RB, or mutated/aberrant P53); WT (low Hec1 protein expression level (< 50% HeLa expression), wild type RB, or wild type P53). 2-tailed t test is utilized to determine significance in P values.

In the same analysis, a higher proportion of wild type P53 cell lines showed more resistance to Hec1 inhibitor TAI-1 compared with those with mutant (including deleted gene) P53 (p < 0.005, Table 4). When the Hec1 expression level was combined with the P53 gene status (wild type vs. mutant/deleted), the correlation was more tight statistically (p < 0.0001, Table 4).

In the analysis of the impact of the RB gene (either hypophosphorylation or deletion), the correlation with response to the Hec1 inhibitor TAI-1 was not established in this database. However, when combined with the Hec1 expression level (dual markers), the correlation with response to TAI-1 was more tight (p < 0.005, Table 4).

When the two markers P53 and RB genes were combined (i.e. the presence of an aberrant P53 and/or RB gene) and correlated with the response to TAI-1, the correlation was also very strong (p < 0.005, Table 4). When combined with the Hec1 expression (i.e. Hec1 expression level combined with the presence of aberrant P53 and/or RB gene), the correlation was very tight (p < 0.0001, Table 4).

### *In vitro* inhibition of RB and P53 and cellular sensitivity to TAI-1

To determine the role of RB and P53 in TAI-1 cellular sensitivity, *in vitro* siRNA knockdown assays were performed in cells carrying wild type RB and P53, respectively. HeLa, which carry mutated RB and mutated P53, was used as the control cell line during the knockdown assays.

**Figure 7:**
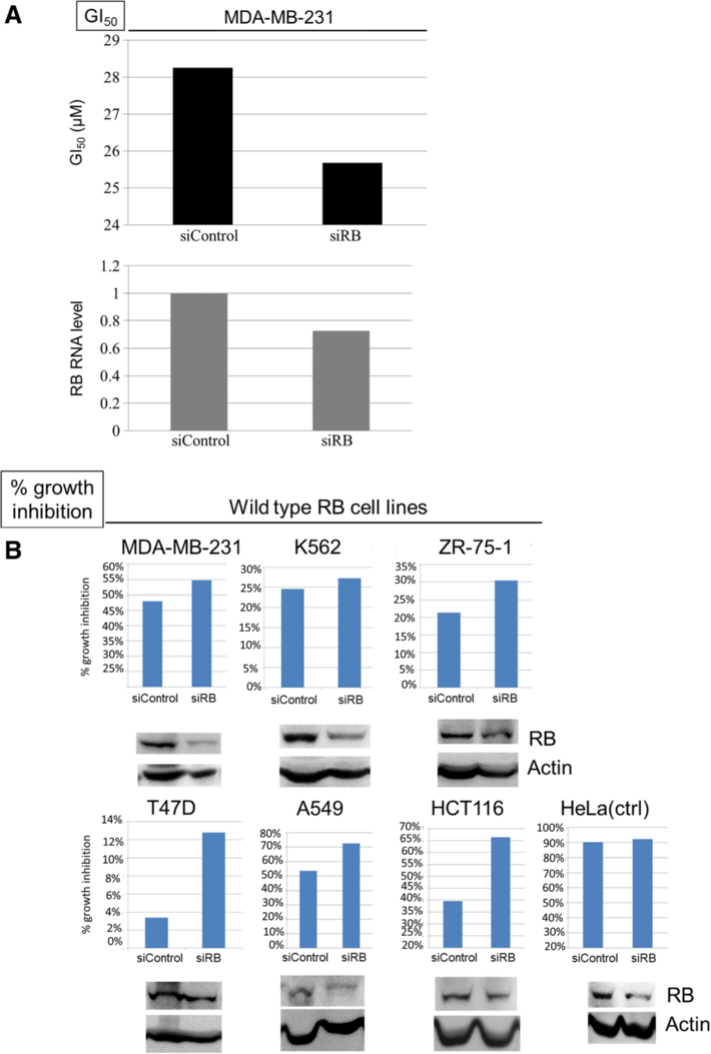
**Efficient knockdown of RB in cancer cells increases cellular sensitivity to TAI-1. (A)** MDA-MB-231 cells which carry wild-type RB were transfected with control siRNA (siControl) or siRNA of RB (siRB) for 24 hours and treated with TAI-1 (starting dose 100 μM, 3x serial dilution), incubated for 48 hours and analyzed for viability with MTS. Cellular sensitivity is expressed in GI_50_ (nM) and RNA from transfected cells were analyzed for RB RNA level by quantitative real time PCR. SiRB reduced GI_50_ of compound in cells. **(B)** Selected cell lines which carry wild type RB (MDA-MB-231, K562, ZR-75-1, T47D, A549, HCT116) or mutated RB (HeLa, as control) were transfected with siRB and treated with TAI-1, incubated for 48 hours and analyzed for viability with MTS. Cellular sensitivity is expressed as% growth inhibition and cell lysates from transfected cells were collected and RB protein levels determined by western blotting. Shown are representative results from at least two independent experiments.

To determine the role of RB in TAI-1 cellular sensitivity, siRNA to RB was used in cell lines carrying wild type RB, including MDA-MB-231, K562, ZR-75-1, T47D, A549, and HCT116. After siRNA treatment, cells were treated with TAI-1 and analyzed at 48 hours after TAI-1 treatment with MTS assay. In the first experiment, a full scale GI_50_ was assessed in MDA-MB-231 cells following siRNA transfection. A 20% decrease in RB RNA levels was seen in conjunction with a 7% decrease of GI_50_ in (Figure 7A). In subsequent experiments with other cell lines (Figure 7B), single dose inhibition was assessed. Using the protocol described in the Methods section, we were able to show the decreased RB protein and this was associated with a 10 ~ 25% enhancement in cancer cell proliferation inhibition (Figure 7B). In experiments with HeLa as a control (known to have RB mutation), siRNA incubation showed a reduction in the expression of the mutant RB but no effect on the cellular sensitivity to TAI-1. To ensure that this effect was not RB-siRNA sequence-specific, knockdown with a different RB-siRNA sequence was conducted which showed similar results (results not shown). Knockdown of RB in wild type RB cancer cells lead to increased sensitivity to TAI-1.

**Figure 8:**
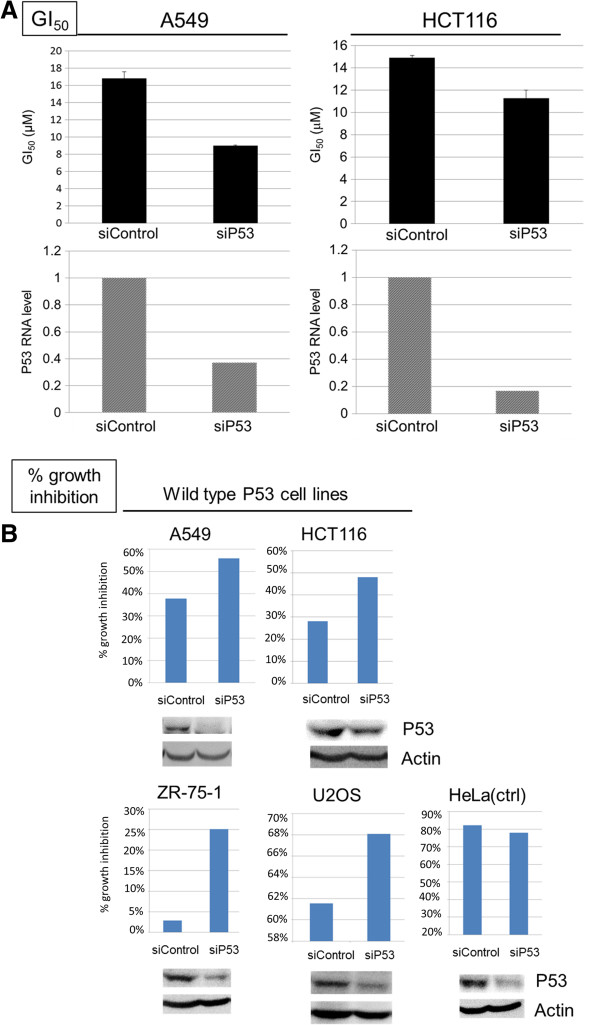
**Efficient P53 knockdown in cancer cells increases cellular sensitivity to TAI-1. (A)** A549 and HCT116 cells which carry wild-type P53 were transfected with control siRNA (siControl) or P53 siRNA (siP53) for 24 hours and treated with TAI-1 (starting dose 100 μM, 3x serial dilution), incubated for 48 hours and analyzed for viability with MTS. Cellular sensitivity is expressed in GI_50_ (nM) and RNA from transfected cells were analyzed for P53 RNA level by quantitative real time PCR. SiP53 reduced GI_50_s of compound in cells. **(B)** Selected cell lines which carry wild type P53 (A549, HCT116, ZR-75-1, U2OS) or mutated P53 (HeLa, as control) were transfected with siP53, treated with TAI-1 and analyzed for viability with MTS. Cellular sensitivity is expressed as% growth inhibition and cell lysates from transfected cells were collected and P53 protein levels determined by western blotting.

To determine the role of P53 in TAI-1 cellular sensitivity, siRNA to P53 was used in cell lines carrying wild type P53, including A549, HCT116, ZR-75-1, and U2OS, were used for P53 knockdown assays. The same methods as RB study were used. As shown in Figure 8A, a 60 ~ 80% decrease in P53 RNA levels lead to 30 ~ 50% decrease of GI_50_ in A549 and HCT116 cells, and this was associated with a 10 ~ 20% increase in the enhancement of cancer cell proliferation inhibition (Figure 8A and B). Again, in HeLa cells, which has a mutant P53 and served as a control, siRNA also inhibit the expression of mutant P53 RNA but had no effect on the cellular proliferation inhibition activity of TAI-1. Furthermore, to ensure that the effect is not siRNA sequence-specific, knockdown with a different P53-siRNA sequence was conducted and showed similar results (results not shown). Knockdown of P53 lead to increased cellular sensitivity to TAI-1 in the cells carrying wild type P53. These results indicate that the status of RB and P53 may affect the activity of Hec1-targeted inhibitor TAI-1 on cancer cells, and cells with a loss of functional RB or P53 may have an increased sensitivity to Hec1-targeted inhibitors.

### Differential Hec1 expression in clinical cancer subtypes

**Figure 9:**
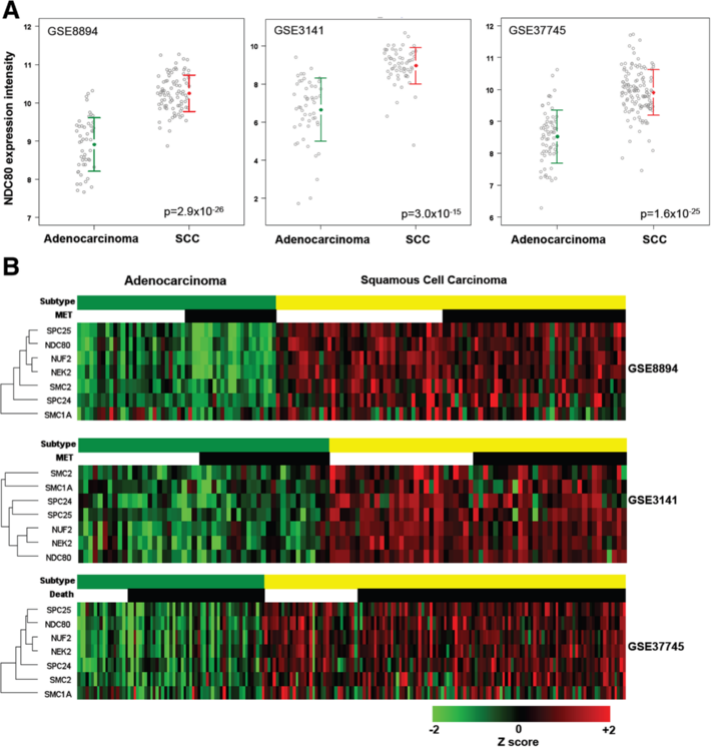
**Differential expression of NDC80 (Hec1) and genes associated with NDC80 between subtypes of non-small cell lung cancer. (A)** NDC80 (Hec1) (Affymetrix Probeset ID 204162_at) expression between adenocarcinoma and squamous cell carcinoma of lung in three different independent datasets (GSE8894, GSE3141 and GSE37745). The unit of Y axis is logarithm of expression intensity to the base 2. ANOVA was used to compare these two subtypes of NSCLC. **(B)** One way hierarchical clustering analysis of NDC80 gene and genes associated with NDC80 for subtypes of NSCLC in the same three independent datasets. The results consistently showed up-regulated expression of NDC80 and its closely associated genes (SPC25, NUF2 and Nek2) in squamous cell carcinoma of lung. Green: adenocarcinoma. Yellow: squamous cell carcinoma. The heat map scale is mean ± 2SD.

Genome-wide expression profile analysis has shown that Hec1 is upregulated in lung, colorectal, liver, breast, and brain tumors and that Hec1 expression correlates with tumor grade and prognosis [4,9]. To determine whether HEC1 expression varies between cancer subtypes from the same tissue or organ, the gene expression data of NDC80 (HEC1) between adenocarcinoma and squamous carcinoma was studied for lung cancer. As shown in Figure 9A, NDC80 expression is significantly higher in squamous cell carcinoma of lung than adenocarcinoma in all three independent datasets. One way hierarchical cluster analysis consistently showed that NDC80, NEK2, NUF2 and SPC25 were reproducibly clustered together in three different gene expression datasets (Figure 9B). All these four genes showed higher expression in squamous cell carcinoma of lung. The results indicate that different subtypes of lung cancer could respond differently to the treatment of Hec1 inhibitor. The predictability of response to Hec1-targeted treatment according to Hec1 associated gene expression remains to be further studied; however, our results suggest such consideration for HEC1 or related gene expression may be an important factor in the design of personalized Hec1-targets treatment of cancers.

## Discussion

This study explored the potential of the improved anticancer agent targeting Hec1 for clinical development and utility. The potency, safety, synergistic effect, markers for response and clinical relevance was evaluated using *in vitro*, *in vivo,* and database analysis methods.

Ever since Hec1 was discovered and characterized, the possibility that this may be a good molecular target was discussed. Hec1 is an oncogene that when overexpressed in transgenic mice leads to tumor formation [5]. The differential expression profile of Hec1 in cancer cells in comparison to normal non-actively dividing cells further supports the suitability of this target for anticancer treatment. The current study shows a small molecule with largely improved potency range enabling the preclinical development of a Hec1 targeted small molecule. The structure-activity relationship is demonstrated for over 200 analogues of the Hec1-targeted small molecule (Huang *et al*, manuscript in preparation).

The improved Hec1-targetd small molecule TAI-1 inhibits the growth of a wide spectrum of cancer cell lines *in vitro*. Interestingly, a small number of cell lines were resistant to TAI-1, suggesting that there may be changes in signaling pathways that allow cells to bypass Hec1 inhibitor induced cell death. This observation prompted our further exploration of markers for TAI-1 response, which may have clinical implications for personalized therapy. A number of known cellular factors were assessed for their impact on the cellular response to TAI-1. The expression of Hec1, its interacting partner RB [29], and P53, a tumor suppressor like RB, were evaluated based on possible crosstalk of pathways. The profile in Table 1 shows a possible association of the status of the tumor suppressors with cellular sensitivity to TAI-1. Analysis of the three factors indicate that the participation of RB is nominal (Table 4), however, the *in vitro* siRNA studies show that RB may play a role in TAI-1 sensitivity (Figure 7). The impact of RB remains to be clarified in future biomarker studies. In contrast, the combined markers Hec1 and P53 showed a significant impact on cellular sensitivity to TAI-1 (Table 4). In addition, the role of P53 is further supported by the *in vitro* siRNA knockdown studies (Figure 8). Although these are very interesting findings, a larger study to allow multivariate analysis will be necessary for more accurate evaluation, but such study is beyond the scope of the current study. Nevertheless, these findings provide a rationale for the building of the parameters for response into future clinical studies for Hec1 inhibitors, in particular TAI-1, and analogues of TAI-1.

In contrast to *in vitro* cell line studies, the *in vivo* models demonstrated efficacy but doesn’t reflect the potency from *in vitro* studies. Administration of drug to animal models, in comparison to cell lines in culture, adds another level of complexity due to possible variability in drug absorption levels due to barriers encountered during oral administration, such as enzymatic degradation, pH sensitivity, drug pumps in the gastrointestinal tract, etc.; hence, the efficacy values between the *in vivo* models and *in vitro* models cannot be directly comparable. It is therefore only appropriate to use these preliminary xenograft models to determine efficacy but not to efficacy doses directly to *in vitro* GI_50_. Furthermore, better comparison of the efficacy doses between xenograft models should be designed so absorption levels are controlled and formulation of the vehicle for administration is optimized. Note that we are the first to evaluate the oral efficacy of Hec1-targeted inhibitors as an anticancer agent and demonstrate efficacy of the improved Hec1-targeted compound in human liver, colon and breast *in vivo* tumor models. Even though the great leap in *in vitro* potency doesn’t correlate well with the *in vivo* efficacy, this study provides a basis for the pharmaceutical development of a Hec1-targeted small molecule based on the significant improvement in *in vitro* efficacy, which translates to a clinically applicable oral dosage. The pharmacological parameters, such as oral absorption, and compound solubility remains to be overcome by further modifications to the core structure and exploration of dosing formulations through the efforts of medicinal chemists and formulation experts.

The safety of TAI-1 was evaluated with activity in normal cell lines, hERG inhibition and a pilot toxicity study. The activity in normal cell lines suggests that TAI-1 has high cancer cell specificity and a high therapeutic index. In combination with hERG inhibition assay, the *in vitro* evaluation shows that TAI-1 is safe as an anticancer agent with little liability on cardiac toxicity. Furthermore, *in vivo* toxicity studies in the same species of mice as the xenograft studies showed no body weight loss and no changes in organ weight and plasma indices. These athymic mice used for *in vivo* modeling were good correlation of the toxicity incurred at efficacy doses in the xenograft models, but were unable to show immunosuppression, a common side effect of chemotherapeutics. In rodent with intact thymus, dosing of TAI-1 lead to a dose-dependent decrease of thymus weights and a slight decrease of spleen weights, but did not showed dose-dependent changes in blood indices, including white blood cells, due to TAI-1 (Additional file 2: Figure S1). It should be noted that it is also possible that the lack of body weight loss and hematological effects may not be evident in only 7 days, and toxicity studies dosed for longer period of times may be able to further determine the long term effects of TAI-1. In contrast to the 7-day toxicity study conducted independently of the xenograft studies in SCID mice, xenograft studies seemed to show a modest body weight loss (up to 13.5% at day 7, n = 6) during dosing (Figure 3). Since this effect was not evident in the independently conducted toxicity studies in the same species of mice (0% change at day 7, n=8), the body weight loss is suggested to be nonspecific to the compound. The body weight loss may be related to the tumor burden or different tumor xenograft interactions, since the change varied between models (11.5% for Huh-7 and 13.5% for Colo205 at day 7). The influencing factors of body weight loss in the xenograft models remains unclear, and further parallel designs of xenograft and toxicity studies may help determine the underlying cause.

The translational implications were further explored with studies in multi-drug resistant (MDR) cell lines, synergistic studies, and clinical databases. The activity in MDR cell lines was shown with other Hec1 analogues (Huang *et al.*, manuscript submitted) and is not specific to the Hec1 analogue TAI-1. The activity in MDR cell lines carry important clinical implications and suggests that Hec1-targeted agents may be able to offered as a treatment option to cancer patients who do not respond to currently available anticancer agents, a major clinical advance. A combinatorial approach incorporating anti-cancer drugs targeting different pathway for treatment regimens is often used to improve medical outcomes. The synergistic effects of TAI-1 with commercial anticancer agents suggest that TAI-1 or its analogues may be very easily incorporated to current multi-drug treatment regimens. A small pilot study using clinical database analysis shows that Hec1 expression may correlate with established patient subtypes, which may further aid in the building of the parameters for response in clinical applications. Further studies in the clinical development of Hec-1 inhibitors will determine whether selection based on these subtypes will aid in the identification of patients who are more likely to respond to Hec1-targeted therapy.

## Conclusion

In conclusion, this study demonstrates the potential of the improved anticancer agent targeting Hec1 for clinical utility. The potency, safety, and translational implications show that a Hec1-targeted small molecule agent can be developed for clinical utility and that a variety of potential clinical applications may be available to support clinical development.

## Abbreviations

Hec1: Highly expressed in cancer protein 1; MDR: Multi-drug resistant

## Competing interests

LYLH, CCC, KJK, JYNL are employees or consultants of Taivex Therapeutics which owns the rights of this compound. YSL, JJH, JMC, SHC, YJT, PYT, CWL, HSL are employees of Development Center of Biotechnology which collaborated with Taivex Therapeutics and will receive royalty of this compound if successfully approved and marketed.

## Authors’ contributions

LYLH carried out the biomarker studies, participated in the design of the cellular, xenograft and toxicology studies, drafted and revised the manuscript. YSL initiated and designed the cell line GI_50_ screening and mechanistic studies. JJH designed and produced the molecule TAI-1. CCC carried out the studies designed by LYL including cell line GI50 screening, synergy, and the apoptotic blots. JMC designed and participated in the animal studies. YJT carried out the toxicology studies. PYT carried out the xenograft studies. SHH produced TAI-1 for the animal studies. KJK concepted and carried out the clinical sample analysis. CWL carried out western blotting studies for Hec1/Nek2 interaction. HSL carried out the chromosome phenotype studies. JYNL initiated, concepted, and participated in the Hec1/Nek2 inhibitor project and did critical revisions of the manuscript. All authors read and approve the final manuscript.

## Supplementary Material

Additional file 1 **Supplementary materials and methods.** This file includes the preparation of TAI-1, supplementary tables for toxicology blood indices, and Additional file 2: Figure S1. Click here for file

Additional file 2: Figure S1. 7-day toxicology study of TAI-1 in rats with intact thymus shows reversible lower thymus and spleen weights and no gastrointestinal changes. Toxicology thymus and spleen weights and gastrointestinal results. Click here for file
