# Supplementary material for: Characterization of the biological activity of a potent small molecule Hec1 inhibitor TAI-1
Source: J Exp Clin Cancer Res. 2014 Jan 9;33(1):6. doi: 10.1186/1756-9966-33-6 (PMC3895848; doi:10.1186/1756-9966-33-6)
Supplement: Additional file 1 — Supplementary materials and methods. This file includes the preparation of TAI-1, supplementary tables for toxicology blood indices, and Additional file 2: Figure S1. [file 1756-9966-33-6-S1.docx]

**SUPPLEMENTARY MATERIALS AND METHODS**

**Preparation of TAI-1 Compound**

The preparation of TAI-1 (N-{4-[4-(4-methoxyphenoxy)-2,6-dimethylphenyl] thiazol-2-yl}isonicotinamide) is illustrated in the following scheme using 1-(4-chloro -2,6-dimethylphenyl)ethanone (**1**) as the starting material. Reaction of compound **1** with 4-methoxyphenol using Pd(OAc)_2_/^t^BuXPhos catalytic system afforded 1-[4-(4-methoxyphenoxy)-2,6-dimethylphenyl]ethanone (**2**) in 75% yield. After reacting with TBABr_3_ and thiourea, **2** was converted to 4-[4-(4-methoxyphenoxy) -2,6-dimethylphenyl]thiazol-2-amine (**3**) in 68% yield. Acylation of **3** by isonicotinoyl chloride afforded *N*-{4-[4-(4-methoxyphenoxy)-2,6-dimethylphenyl] thiazol-2-yl}isonicotinamide (**TAI-1**) in 95% yield. Spectroscopic data for TAI-1: ^1^H NMR (500 MHz, CDCl_3_) δ 8.73 (m, 2 H), 7.62 (m, 2 H), 6.90–6.96 (m, 4 H), 6.80 (s, 1 H), 6.45 (s, 2 H), 3.83 (s, 3 H), 1.92 (s, 6 H); ESI-MS: *m*/*z* 431.7 (M + H)^+^.

**Scheme**

*^a^*Reagents and conditions: (i) 4-methoxyphenol, Pd(OAc)_2_, ^t^BuXPhos, toluene, 100 °C, 75%. (ii) (a) TBABr_3_, CH_3_CN, r.t.; (b) thiourea, EtOH, reflux; 68% (two steps). (iii) isonicotinoyl chloride, DMAP, CH_2_Cl_2_, 95%.

Experimental Details are as follows: **1-[4-(4-Methoxyphenoxy)-2,6-dimethylphenyl]ethanone (2).** To a solution of 1 (10.0 g, 54.8 mmol), K3PO4 (23.2 g, 110 mmol) 4-methoxyphenol (8.16 g, 65.7 mmol) in toluene (78.2 mL), 2-di-tert-Butylphosphino-2',4',6'-triisopropylbiphenyl (tBuXPhos, 349 mg, 0.82 mmol) and Pd(OAc)2 (259 mg, 1.15 mmol) were added. The reaction was heated at 100°C for 5.0 h under N_2_. The solution was cooled to room temperature and filtered through a small pad of Celite. The cake was washed with EtOAc (50 mL) and combined filtrate was concentrated under reduced pressure. The residue was recrystallized in MeOH to give **2** (11.1 g) as white solids in 75% yield: ^1^H NMR (CDCl_3_) δ 6.96 (d, *J* = 9.2 Hz, 2 H), 6.88 (d, *J* = 9.2 Hz, 2 H), 6.57 (s, 2 H), 3.81 (s, 3 H), 2.46 (s, 3 H), 2.20 (s, 6 H); ESI-MS: *m*/*z* 271.1 (M + H)^+^.

**4-[4-(4-Methoxyphenoxy)-2,6-dimethylphenyl]thiazol-2-amine (3).** To a solution of **2** (3.80 g, 14.1 mmol) in acetonitrile (28.1 mL), tetrabutylammoniumtribromide (TBABr_3_, 7.46 g, 15.5 mmol) was added. The reaction was stirred at room temperature overnight. The solution was concentrated under reduced pressure, added with water, and extracted with EtOAc. The organic layer was washed with brine, dried over anhydrous MgSO_4_(s), and concentrated under reduced pressure. The residue (4.90 g, 14.0 mmol) was added with thiourea (1.07 g, 14.1 mmol) in 95% EtOH (20.0 mL). The reaction mixture was heated at reflux for 100 min. The solution was concentrated and added with water (100 mL) and saturated aqueous Na_2_CO_3_ (5.0 mL). The resultant precipitate was filtered and recrystallized in toluene. The solids were filtered and dried under vacuum to give **3** (3.10 g) as yellow solids in 68% yield (two steps): ^1^H NMR (CDCl_3_) δ 6.98 (d, *J* = 9.0 Hz, 2 H), 6.88 (d, *J* = 9.0 Hz, 2 H), 6.64 (s, 2 H), 6.27 (s, 1 H), 5.40 (bs, 2 H), 3.81 (s, 3 H), 2.13 (s, 6 H); ESI-MS: *m*/*z* 327.1 (M + H)^+^.

***N*-{4-[4-(4-Methoxyphenoxy)-2,6-dimethylphenyl]thiazol-2-yl}isonicotinamide (TAI-1).** To a solution of **3** (2.00 g, 6.13 mmol) in CH_2_Cl_2_ (15.3 mL) were added DMAP (2.25 g, 18.4 mmol) and isonicotinoyl chloride hydrochloride (1.64 g, 9.21 mmol). The reaction mixture was stirred at room temperature overnight. The solution was concentrated under reduced pressure and added with water. The resultant precipitate was filtered, and recrystallized in toluene to give **TAI-1** (2.50 g) as light-yellow solids in 95% yield: ^1^H NMR (CDCl_3_) δ 8.71 (d, *J* = 6.0 Hz, 2 H), 7.60 (d, *J* = 6.0 Hz, 2 H), 6.90–6.96 (m, 4 H), 6.79 (s, 1 H), 6.40 (s, 2 H), 3.83 (s, 3 H), 1.90 (s, 6 H); ESI-MS: *m*/*z* 431.7 (M + H)^+^.

**Pilot Toxicology Study in Rats**

A sub-acute toxicology study was performed for TAI-1. Female SD rats (7 weeks old) were used in this study. Rats were divided into four treatment groups: vehicle control (20% hydroxypropyl-beta-cyclodextrin (HPβCD)), test article (in vehicle) at 10, 25, and 50 mg/kg, and all rats were treated twice a day by oral administration for 7 days (n = 6 for each group). After drug withdrawal, rats were allowed 14 days of recovery period. Organ weights were measured on day 7 and 21

**Table S1.** Red Blood Cell Indices of athymic SCID mice

|  | Vehicle | | TAI-1 7.5 mpk | | TAI-1 22.5 mpk | | TAI-1 75 mpk | |
| --- | --- | --- | --- | --- | --- | --- | --- | --- |
|  | Mean | SEM | Mean | SEM | Mean | SEM | Mean | SEM |
| RBC (10^4^/μL) | 121.9 | 41.9 | 146.1 | 22.4 | 93.1 | 11.8 | 108.6 | 13.5 |
| HGB (g/L) | 9.7 | 0.3 | 9.9 | 0.1 | 9.8 | 0.2 | 9.6 | 0.7 |
| HCT (%) | 156.4 | 2.7 | 159.8 | 2.6 | 156.0 | 3.1 | 154.1 | 10.2 |
| MCV (fL) | 43.4 | 1.1 | 43.9 | 0.5 | 44.0 | 0.9 | 43.7 | 2.9 |
| MCH (pg) | 44.9 | 0.1 | 44.4 | 0.2 | 44.7 | 0.3 | 45.9 | 0.4 |
| MCHC (g/L) | 16.2 | 0.2 | 16.2 | 0.1 | 15.9 | 0.1 | 16.2 | 0.1 |
| PLT (10^3^/μL) | 360.9 | 5.2 | 364.0 | 2.2 | 354.9 | 2.2 | 352.8 | 3.2 |
| RDW-SD (fL) | 251.0 | 50.0 | 160.8 | 45.0 | 292.5 | 75.3 | 213.0 | 53.2 |
| RDW-CV (%) | 20.1 | 0.5 | 21.6 | 0.8 | 20.1 | 0.7 | 22.1 | 0.8 |

**Table S2.** White Blood Cell Indices of athymic SCID mice

|  | Vehicle | | TAI-1 7.5 mpk | | TAI-1 22.5 mpk | | TAI-1 75 mpk | |
| --- | --- | --- | --- | --- | --- | --- | --- | --- |
|  | Mean | SEM | Mean | SEM | Mean | SEM | Mean | SEM |
| WBC (10^3^/μL) | 1.22 | 0.42 | 1.46 | 0.22 | 0.93 | 0.12 | 1.09 | 0.13 |
| Neutrophil (10^3^/μL) | 0.44 | 0.09 | 0.31 | 0.08 | 0.53 | 0.05 | 0.40 | 0.09 |
| Lymphocyte (10^3^/μL) | 0.17 | 0.03 | 0.24 | 0.04 | 0.17 | 0.05 | 0.11 | 0.03 |
| Monocyte (10^3^/μL) | 0.01 | 0.00 | 0.03 | 0.01 | 0.02 | 0.01 | 0.03 | 0.01 |
| Eosinophil (10^3^/μL) | 0.19 | 0.04 | 0.50 | 0.09 | 0.20 | 0.06 | 0.29 | 0.11 |
| Basophil (10^3^/μL) | 0.09 | 0.02 | 0.10 | 0.03 | 0.04 | 0.01 | 0.08 | 0.03 |
| Neutrophil (%) | 47.13 | 8.16 | 27.52 | 6.98 | 58.49 | 5.10 | 49.69 | 7.46 |
| Lymphocyte (%) | 22.81 | 5.06 | 20.23 | 2.83 | 16.34 | 3.18 | 12.07 | 1.58 |
| Monocyte (%) | 1.91 | 0.60 | 2.50 | 1.02 | 2.59 | 0.59 | 3.21 | 0.85 |
| Eosinophil (%) | 24.87 | 5.69 | 40.70 | 7.83 | 19.13 | 3.06 | 27.24 | 6.22 |
| Basophil (%) | 8.56 | 1.86 | 7.83 | 2.24 | 3.50 | 0.92 | 9.05 | 2.43 |
