# Supplementary figures and images for: Characterization of the biological activity of a potent small molecule Hec1 inhibitor TAI-1
Source: J Exp Clin Cancer Res. 2014 Jan 9;33(1):6. doi: 10.1186/1756-9966-33-6 (PMC3895848; doi:10.1186/1756-9966-33-6)

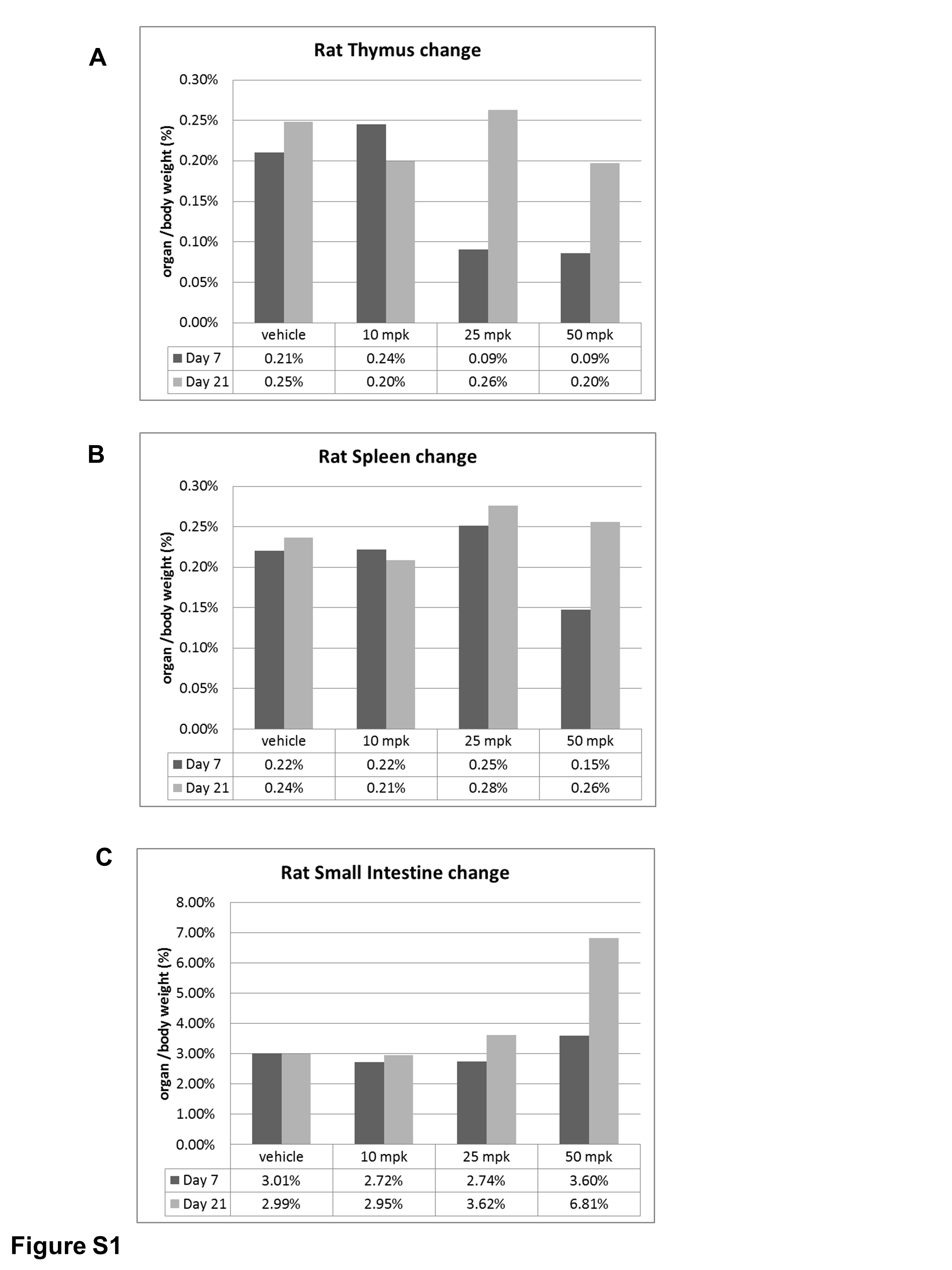

Supplement: Additional file 2: Figure S1. — 7-day toxicology study of TAI-1 in rats with intact thymus shows reversible lower thymus and spleen weights and no gastrointestinal changes. Toxicology thymus and spleen weights and gastrointestinal results. [file 1756-9966-33-6-S2.tiff]
